# Development and Validation of a Scaffold‐Free Human Multilineage Spheroid Model for Early Stage Cholangiopathies Driven by Cholangiocyte Senescence

**DOI:** 10.1111/liv.70352

**Published:** 2025-09-10

**Authors:** Cheuk‐Ting Wu, Anja Moncsek, Joachim Mertens

**Affiliations:** ^1^ Institute of Anatomy, University of Bern Bern Switzerland; ^2^ Department of Quantitative Biomedicine University of Zurich Zurich Switzerland; ^3^ GastroZentrum Hirslanden, Digestive Disease Center Zürich Switzerland

**Keywords:** Bcl‐xL inhibitor, cholangiocyte senescence, hepatobiliary disease, in vitro 3D culture model, liver fibrosis

## Abstract

**Background and Aims:**

Cholangiopathies, including primary sclerosing cholangitis (PSC), primary biliary cholangitis (PBC), and post‐COVID‐19 cholangiopathy (PCC), involve chronic cholangiocyte injury, senescence, epithelial–stromal crosstalk, and progressive fibrosis. However, effective in vitro models to capture these interactions are limited. Here, we present a scaffold‐free 3D multilineage spheroid model, composed of hepatocyte‐like cells (HepG2), cholangiocytes (H69), and hepatic stellate cells (LX‐2), designed to recapitulate early fibrogenic responses driven by senescent cholangiocytes.

**Methods:**

Cholangiocyte senescence was induced via ionising radiation prior to spheroid assembly. Fibrosis progression was assessed by mRNA expression of hepatic stellate cell (HSC) activation markers, immunofluorescence, and Masson's trichrome staining, in both direct HSC co‐culture and spheroids. To evaluate therapeutic potential, spheroids were treated with A‐1331852, a selective Bcl‐xL inhibitor known to induce apoptosis in senescent cells in PSC and PBC mouse models Drug efficacy was measured by apoptosis induction, reduction of fibrosis, and modulation of stromal activation.

**Results:**

Senescent cholangiocytes induced robust HSC activation and promoted extracellular matrix (ECM) deposition, mimicking early fibrogenesis. Treatment with A‐1331852 selectively induced apoptosis in senescent cholangiocytes and activated HSCs, leading to a marked reduction in fibrosis, consistent with murine PSC and PBC models.

**Conclusions:**

This 3D multilineage spheroid model offers a mechanistically relevant and scalable platform to study cholangiocyte senescence‐driven fibrosis. Its applicability to senolytic drug testing supports its use in preclinical screening and translational research across a spectrum of cholangiopathies.

**Abbreviations**
3D: three‐dimensional
Bcl‐2: B‐cell lymphoma 2
Bcl‐xL: B‐cell lymphoma‐extra‐large
BH3: Bcl‐2 homology domain 3
BSA: bovine serum albumin
CCA: cholangiocarcinoma
c‐H69: non‐irradiated, control, H69 cells with respect to the irradiated H69
CK19: cytokeratin 19
COL1A1: alpha‐1 type I collagen
COL3A1: alpha‐1 type III collagen
DAPI: 40,6‐diamidino‐2‐phenylindole
ECM: extracellular matrix
HSC: hepatic stellate cell
IHC: immunohistochemistry
IR: ionising radiation
*Mdr2* ^*−/−*^
: multidrug resistance 2 gene knockout
PDGF: platelet‐derived growth factor
PSC: primary sclerosing cholangitis
RIPK3: receptor interacting serine/threonine kinase 3
SASP: senescence‐associated secretory phenotype
s‐H69: senescent H69 cells harvested 4 days after irradiation
TIMP‐1: tissue inhibitor of metalloproteinase 1
α‐SMA: alpha‐smooth muscle actin

**Summary**

- Our study introduces a novel 3D cell culture model designed to investigate how senescent cholangiocytes, bile duct epithelial cells that have entered a state of irreversible stress, contribute to liver fibrosis.
- By incorporating senescent cholangiocytes, HSCs, and hepatocyte‐like cells in a scaffold‐free spheroid, this model captures key early features of cholangiopathies such as PSC, PBC, PCC and DIC.
- This simplified yet powerful system allows researchers to explore how bile duct cell damage triggers fibrotic signalling in surrounding liver tissue.
- It also enables testing of potential therapies that specifically target senescent cholangiocytes, offering a promising path toward more effective and targeted treatments for chronic liver diseases.
- By reducing the need for animal experiments, this model provides a faster, more ethical, and cost‐effective approach to studying complex liver disorders.

## 1 Introduction

Cholangiocytes, the epithelial cells lining the bile ducts, are increasingly recognised as central players in a broad spectrum of liver diseases. Once considered passive conduits for bile transport, they are now known to actively regulate inflammation, fibrosis, and liver development and regeneration [1, 2, 3]. The structure and function of cholangiocytes can be disrupted by a range of insults, such as genetic mutations, infections, immune‐mediated factors, xenobiotics and even idiopathic causes, leading to cholestasis and biliary dysfunction [1, 4, 5].

Cholangiocyte dysfunction is central to classical cholangiopathies such as PSC [6], PBC [7] and PCC [8]. Dysregulated cholangiocytes contribute to ductular reaction, and fibrogenic signalling, ultimately driving progressive fibrosis, cirrhosis, and, in some cases, hepatocellular carcinoma (HCC) or cholangiocarcinoma (CCA) [1].

Among the maladaptive responses to chronic liver injury, cellular senescence in cholangiocytes has emerged as a critical mechanism in fibrotic progression. Senescence is a prolonged growth arrest triggered by sustained stress, DNA damage, or metabolic dysfunction, and is characterised by the senescence‐associated secretory phenotype (SASP), a paracrine programme of pro‐inflammatory and pro‐fibrotic signalling. These senescent cholangiocytes persist within tissue and actively contribute to hepatic stellate cell (HSC) activation and periductular fibrosis through SASP factors such as TGF‐β, IL‐6 and MMPs [3, 4, 9, 10].

Reactive ductular cholangiocytes, initially proliferative, can evolve into senescent cells under unresolved injury, representing a pathological inflection point from adaptive regeneration to chronic fibrosis [1, 11]. Senescent cholangiocytes have been observed in prolonged stages of PSC, PBC, NASH and chronic viral hepatitis, and their spatial proximity to α‐SMA+ myofibroblasts and collagen deposition highlights their role in fibrogenesis [7, 9, 11, 12]. In PSC, this process may contribute to the formation of ‘onion‐skin’ fibrosis, and prolonged SASP exposure may even promote CCA initiation [13, 14, 15, 16].

Despite advances in 3D culture systems for modelling cholangiopathies [17], experimental models that allow mechanistic investigation of the crosstalk between senescent cholangiocytes and HSCs remain limited. Traditional 2D monolayer cultures fail to replicate the multicellular architecture and dynamic cell–cell interactions of liver tissue, while in vivo models are resource‐intensive and often less manipulable. A physiologically relevant, scalable, and reductionist 3D platform is needed to study senescence‐driven fibrogenesis and enable drug screening targeting epithelial–stromal crosstalk.

Here, we present a 3D co‐culture spheroid model consisting of human cholangiocytes (H69), hepatic stellate cells (HSCs), and hepatocyte‐like cells (HepG2) to investigate the fibrogenic consequences of cholangiocyte senescence. The model reproduces key features of cholangiopathies, including fibrotic remodelling and HSC activation, in a controlled setting. To demonstrate translational relevance, we treated senescent spheroids with A‐1331852, a Bcl‐xL inhibitor previously shown to induce apoptosis in senescent cholangiocytes in PSC and PBC mouse models [9, 18]. Consistent with prior in vivo findings, A‐1331852 selectively eliminated senescent cholangiocytes in our model, validating its use as a platform for senolytic screening and advancing understanding of fibrogenic epithelial–mesenchymal signalling in cholestatic liver diseases.

## 2 Materials and Methods

### 2.1 Cell Lines

HepG2 cells were purchased from Sigma Aldrich and cultured in Dulbecco's modified Eagle's medium (DMEM) supplemented with 1 g/L glucose, 4 mM l‐glutamine, 1 mM sodium pyruvate and 20% fetal bovine serum (FBS). The human HSC cell line LX2, generously provided by Dr. S. Friedman (Icahn School of Medicine at Mount Sinai, USA), was cultured in DMEM supplemented with 4.5 g/L glucose, 4 mM l‐glutamine, 1 mM sodium pyruvate and 10% fetal bovine serum (FBS). H69 cells, provided by PD Dr. M. Sinnreich (Department of Biomedicine, University of Basel, Switzerland), were maintained in a medium containing DMEM (1 g/L glucose): DMEM/F12 (1:1) supplemented with 10% FBS, adenine (24.3 μg/mL), insulin (5 μg/mL), epinephrine (1 μg/mL), triiodothyronine (2.26 ng/mL), transferrin (8.3 μg/mL), epidermal growth factor (EGF) (10 ng/mL) and hydrocortisone (0.56 μg/mL). All cells were cultured at 37°C in a humidified atmosphere containing 5% CO_2_.

### 2.2 Spheroid Culture

#### 2.2.1 Bi‐Cellular Spheroid Formation

To specifically investigate the direct interaction between cholangiocytes and hepatic stellate cells, bi‐cellular spheroids consisting of H69 and LX2 cells were generated. Homogenised cells in a 1:2 ratio (H69:LX2) were seeded in Terasaki plates Greiner Bio‐One at a density of 2000 viable cells in 20 μL medium per well. The culture medium is DMEM (1 g/L glucose) supplemented with 4 mM l‐Glutamine and 1 mM Sodium Pyruvate, containing 10% DMEM/F12, 15% FBS, 5 μg/mL adenine, 1 μg/mL insulin, 0.2 μg/mL epinephrine, 0.5 ng/mL triiodothyronine, 1.66 μg/mL transferrin, 2 ng/mL epidermal growth factor and 0.11 μg/mL hydrocortisone. The spheroids were co‐cultured using the hanging drop method. Spheroids were incubated for 24 h at 37°C in a humidified atmosphere with 5% CO_2_. These bi‐cellular spheroids were primarily used for subsequent gene expression analysis.

#### 2.2.2 LX‐2 Spheroid Formation for Activation Induced by PDGF‐BB

As a positive control of HSC activation gene analysis, LX‐2 only spheroids were generated at a total density of 2000 viable cells per spheroid using the hanging drop method and the same condition as described above.

#### 2.2.3 Multilineage Spheroid Formation

To generate spheroids, homogenised cells were seeded into Terasaki plates (Greiner Bio‐One) at a density of 2000 viable cells in 20 μL medium per well. Spheroids composed of HepG2, LX2 and H69 cells in a ratio of 14:2:1 were cultured in DMEM (1 g/L glucose) supplemented with 4 mM l‐Glutamine and 1 mM Sodium Pyruvate, containing 10% DMEM/F12, 15% FBS, 5 μg/mL adenine, 1 μg/mL insulin, 0.2 μg/mL epinephrine, 0.5 ng/mL triiodothyronine, 1.66 μg/mL transferrin, 2 ng/mL epidermal growth factor and 0.11 μg/mL hydrocortisone. After 3 days, spheroids were transferred to 48‐well plates pre‐coated with Anti‐Adherence Rinsing Solution (Stemcell technologies) with fresh medium and cultured for up to 10 days. Spheroids were maintained at 37°C in a humidified atmosphere with 5% CO_2_.

### 2.3 Gene Expression Analysis qPCR

For mRNA expression analysis, 10 spheroids were pooled to generate one biological replicate. Each *n* value therefore represents RNA extracted from an independent pool of 10 spheroids. The number of biological replicates per condition is indicated in figure legends.

RNA extraction was performed using the Maxwell RSC simply RNA Tissue Kit with the Maxwell RSC Instrument (Promega) according to the manufacturer's instructions. The concentration of extracted RNA was quantified using the NanoDrop One/OneC Microvolume UV–Vis Spectrophotometer (ThermoFisher Scientific). Subsequently, 500 ng of total isolated RNA was reverse transcribed into complementary DNA (cDNA) using the High‐Capacity cDNA Reverse Transcription Kit (Applied Biosystems) according to the manufacturer's protocol. Quantitative real‐time PCR (qPCR) was performed using TaqMan Fast Advanced Master Mix (Applied Biosystems). TaqMan gene expression assays targeting ACTA2 (Hs00426835_g1), COL1A1 (Hs00164004_m1), TIMP‐1 (Hs00171558_m1), COL3A1 (Hs00943809_m1) and HPRT1 (Hs01003268_g1) were purchased from Thermo Fisher Scientific. Gene expression levels were normalised to the housekeeping gene HPRT1 (Hs01003268_g1) and calculated relative to the Day 0 timepoint within each experimental group (normal or senescent spheroids) using the 2^−∆∆CT^ method.

### 2.4 Cell Viability

The cell viability of spheroids was assessed using the CellTiter‐Glo 3D Cell Viability Assay (Promega), which measures luminescent ATP levels according to the manufacturer's instructions. One spheroid was used per reaction. For the positive control, spheroids were pre‐incubated in 70% ethanol for 15 min prior to analysis.

### 2.5 Masson's Trichrome Staining and Immunofluorescence Staining

Spheroids were fixed in 4% formalin for 15 min at room temperature, washed twice with PBS, and embedded in HistoGel (Richard‐Allan Scientific) according to the manufacturer's protocol. The embedded spheroids were processed through an automated tissue processor (Leica TP 1020) for dehydration and paraffin saturation. The processed gel blocks containing fixed spheroids were then embedded in paraffin and sectioned using a microtome (Zeiss, HYRAX M15). Masson's trichrome staining was performed on 3 μm sections, and immunofluorescence staining was performed on 5 μm sections.

For Masson's trichrome staining, the Trichrome Stain (Masson) Kit (Sigma Aldrich) was used according to the manufacturer's protocol. Quantification of collagen signal was performed using ImageJ software, as described by Xu et al. [19]. The percentage of fibrosis‐positive area was calculated by dividing the area stained blue (representing collagen fibres) by the total area of the spheroid.

For immunofluorescence staining, sections were deparaffinised, rehydrated through a series of alcohol dilutions, and then incubated in target retrieval solution (pH 6 or pH 9, depending on the specific antibody requirements, Dako) at 98°C for 30 min. After cooling, the sections were washed three times with PBS and then incubated with 10% bovine serum albumin (BSA, Sigma Aldrich) in PBS for 1 h. The sections were then incubated with diluted primary antibodies (listed in Table S1) in 3% BSA overnight at 4°C. After three washes with PBS, sections were incubated with secondary antibodies and DAPI diluted in 3% BSA for 1 h at room temperature. Finally, slides were mounted with fluorescent mounting medium (Dako) and images were captured using Zeiss Axio Imager Z2 with ZEN software. Quantification of co‐signal and signal area was performed using ImageJ. The area fraction was calculated by dividing the respective signals by the total area of the spheroid.

### 2.6 Senescence Induction

H69 cells were seeded at a density of 3.13 × 10^4^ cells/cm^2^ and incubated overnight at 37°C in a humidified atmosphere containing 5% CO_2_ [18, 20]. The cells were then exposed to 10 Gy of γ‐irradiation and the medium was replaced with fresh medium. The irradiated cells were then cultured for 4 days to achieve a maximum percentage of senescence (40%–50%), as determined by senescence‐associated β‐galactosidase (SA‐β‐gal) staining, before being incorporated into spheroids.

### 2.7 Senescence‐ Associated β‐Galactosidase (SA‐β‐Gal) Staining

Senescence was assessed using the Senescence β‐galactosidase Staining Kit (Cell Signalling) according to the manufacturer's instructions. Cells showing blue signals after β‐gal staining were considered senescent.

For flow cytometry analysis, the CellEvent Senescence Green Flow Cytometry Assay Kit (ThermoFisher Scientific) was used according to the manufacturer's protocol. Analysis was performed using an LSR II Fortessa flow cytometer and data were analysed using FlowJo software.

### 2.8 Western Blot

Spheroids were washed with PBS and lysed in M‐Per Mammalian Protein Extraction Reagent (ThermoFisher Scientific) supplemented with dissolved cOmplete Protease Inhibitor cocktail (Sigma Aldrich) for 30 min on ice. Protein concentration was determined by NanoDrop One/OneC Microvolume UV–Vis Spectrophotometer (ThermoFisher Scientific) and 10 to 20 μg protein per sample was denatured at 95°C with 10% volume of 500 mM DTT and NuPAGE LDS Sample Buffer (4×) (ThermoFisher Scientific). Equal volumes of lysate were subjected to SDS‐PAGE and then transferred to 0.45 μm PVDF membranes. Membranes were then blocked with 10% BSA for 1 h at room temperature and subsequently with primary antibodies diluted in 3% BSA overnight at 4°C. After incubation with horseradish peroxidase‐conjugated secondary antibodies for 1 h at room temperature, proteins were visualised using an ECL chemiluminescence detection substrate (PerkinElmer).

All antibodies used are listed in the Table S2.

### 2.9 Statistical Analysis

Data represent at least three independent experiments (biological replicates), and are described as mean ± standard deviation (SD). For qPCR data, each data point derived from a pooled sample of 10 spheroids to ensure sufficient material for analysis. Quantitative data were analysed, and graphs were generated using GraphPad Prism software (version 8; GraphPad Software). Statistical significance was determined using unpaired, two‐tailed t‐tests. Symbols indicate statistical significance as follows: *p < 0.05, **p < 0.01, ***p < 0.001.

## 3 Results

### 3.1 Viability and Cellular Integrity of Human Multilineage Control Liver Spheroid (c‐H69/LX‐2/HepG2) Maintained for 10 Days in Culture

**FIGURE 1:**
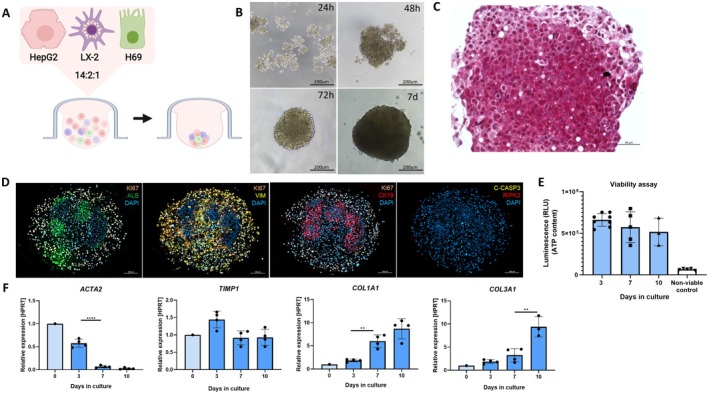
Set‐up and Stability of control human multilineage liver spheroids (c‐H69/LX‐2/HepG2). (A) Schematic overview of the composition and formation of the liver spheroid containing hepatocytes (HepG2), HSCs (LX‐2) and non‐irradiated cholangiocytes (c‐H69). Spheroids were generated using the hanging drop method and maintained in culture for up to 10 days. Illustration created with BioRender.com. (B) Bright field images of spheroids taken at different times during the 7‐day culture period. (C) Masson's trichrome staining of spheroids at Day 10. (D) Immunofluorescence co‐staining of 10‐day‐old spheroids combining proliferation marker Ki67 and cell specific markers (Albumin, Vimentin and CK19), and cell death markers (cleaved caspase 3 and RIPK3). (E) The viability of the spheroids over time in culture was assessed using an ATP‐based viability assay. (F) Relative mRNA expression of HSC activation markers including ACTA2, COL1A1, TIMP‐1 and COL3A1 expression in control and senescent spheroids at days 0, 3, 7 and 10. Expression is shown as fold change relative to Day 0, normalised to HPRT. Each point represents a pool of 10 spheroids; data are mean ± SD of at least three independent experiments (**p < 0.01, ****p < 0.0001).

Control human multilineage liver spheroids consisting of non‐irradiated H69 (c‐H69), LX‐2 and HepG2 cells in a physiological ratio of 1:2:14 were generated using the hanging drop method, resulting in reproducible 3D spheroid formation (Figure 1A). Cell aggregation began within 24 h post‐seeding, and by 72 h, cells had formed compact spheroids of approximately 300 μm in diameter, which remained morphologically stable for up to 7 days in culture (Figure 1B). Masson's trichrome staining revealed no detectable blue collagen deposition, indicating the absence of fibrotic ECM (Figure 1C). Immunofluorescence co‐staining of proliferation marker Ki67 with lineage‐specific markers: albumin for hepatocytes, vimentin for HSCs, and cytokeratin 19 (CK19) for cholangiocytes, confirmed the distribution and proliferation of each cell type within the spheroids after 10 days. The presence of albumin protein further indicated hepatocyte functionality, while cleaved caspase 3 and RIPK3 staining indicated minimal cell death within the spheroids. Spheroid viability was confirmed by ATP quantification over the culture period (Figure 1D). qPCR analysis revealed low initial expression of HSC activation markers (ACTA2, TIMP1, COL1A1, and COL3A1) in the first 3 days, with a gradual upregulation of ECM genes (COL1A1 and COL3A1) over the culture period, likely reflecting matrix accumulation during spheroid growth and structural remodelling rather than fibrotic activation [21, 22] (Figure 1E).

Together, these findings confirm that the hepatocytes, HSCs, and cholangiocytes within the control human multilineage liver spheroids (H69/LX‐2/HepG2) maintain cell‐type identity, functional viability, and architectural stability over a 10‐day culture period without external stimuli. This system provides a reliable foundation for the subsequent induction of senescence‐associated fibrotic phenotypes.

### 3.2 Senescent Cholangiocytes Induce HSC Activation in 3D Spheroids

**FIGURE 2:**
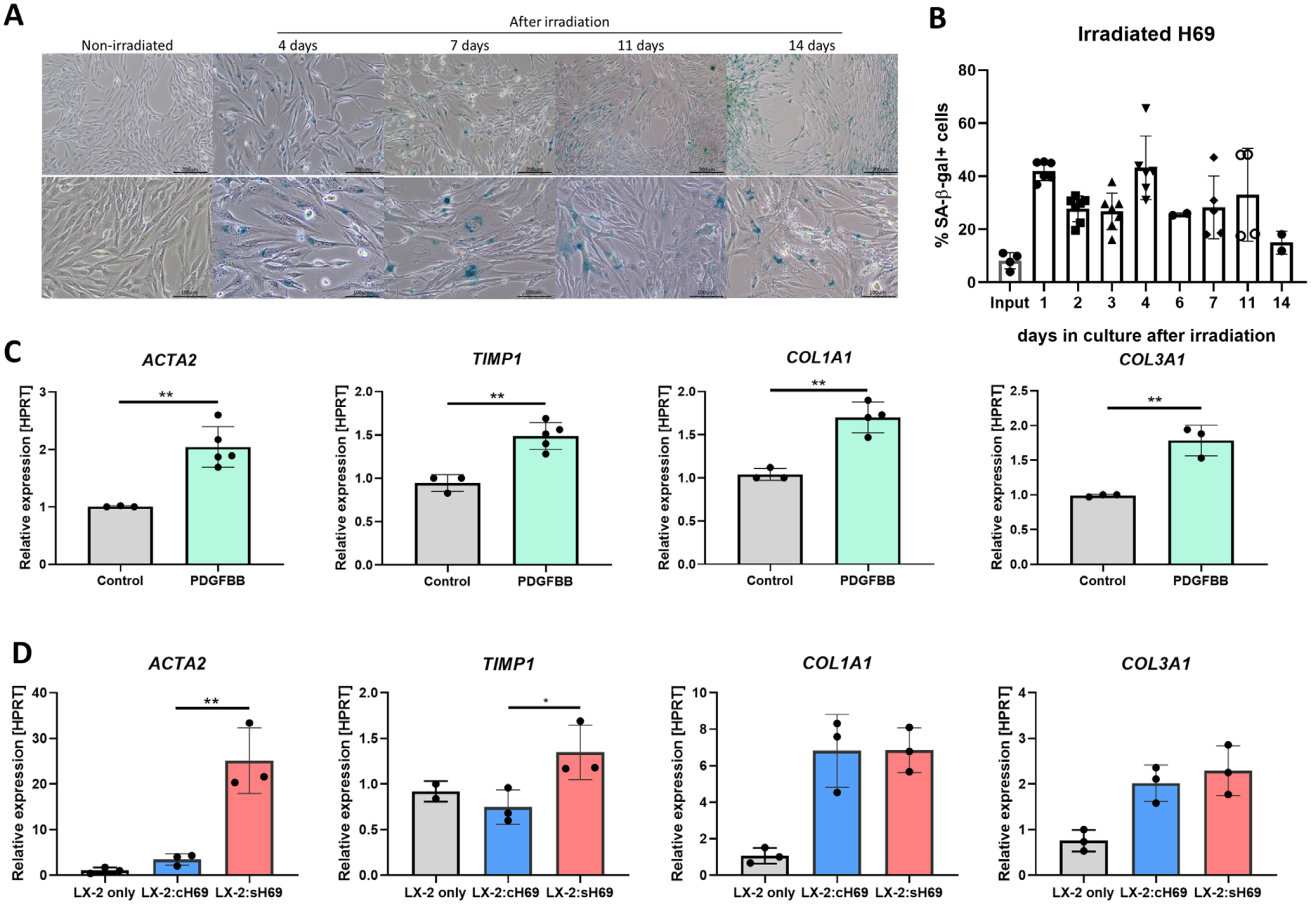
Characteristics of irradiation‐induced senescence in H69 and HSC activation in LX‐2 by PDGF‐BB and co‐culture with senescent H69. (A) β‐Galactosidase staining of c‐H69 and γ‐irradiated H69 cells to detect SA‐β‐gal activity, indicated by blue signals. (B) Flow cytometry analysis of β‐galactosidase‐positive cells in γ‐irradiated H69 cells cultured in 2D, showing the percentage of senescent cells over time. γ‐irradiated H69 cells harvested 4 days after irradiation were then regarded as senescent H69 (s‐H69) cells. (C) qPCR analysis of HSC activation markers in LX‐2 spheroids treated with 50 ng/mL PDGF‐BB for 24 h. The expression of HSC activation markers was normalised to HPRT (**p < 0.01). Data are fold change relative to control, normalised to HPRT. Each data point represents pooled spheroids (*n* = 10). Mean ± SD from at least three independent experiments. Statistical significance: *p < 0.05, **p < 0.01, ***p < 0.001, ****p < 0.0001. (D) qPCR analysis of HSC activation markers in LX‐2 spheroids co‐cultured with c‐H69 and s‐H69 cells, respectively. The expression of HSC activation markers was normalised with HPRT. (*p < 0.05, **p < 0.01). Data are fold change relative to LX‐2 only control, normalised to HPRT. Each data point represents pooled spheroids (*n* = 10). Mean ± SD from at least three independent experiments. Statistical significance: *p < 0.05, **p < 0.01, ***p < 0.001, ****p < 0.0001.

Persistent chronic injury experienced by cholangiocytes in various immune‐related cholangiopathies induces cellular senescence [3, 6, 10]. Cholangiocyte senescence contributes to the pathogenesis of cholangiopathies, mainly PBC and PSC, and is regarded as a key pathogenic process [6, 23, 24]. In PSC, it is recognised as an early event and persists throughout all stages of PSC and is associated with clinical severity in patients [6]. Expression of senescence markers in other cholangiopathy and hepatic disease increases as the diseases advance [11]. To induce senescence, H69 cells were irradiated with 10 Gy γ‐irradiation as previously described [18, 20], resulting in detectable senescence in cultured H69 cells for up to 14 days, as confirmed by SA‐β‐Gal staining (Figure 2A). Quantification by flow cytometry showed a stable percentage of H69 cells expressing β‐gal, indicating the senescent state, averaging 30% over 11 days, with the highest percentage (40%–50%) detected after 4 days. The γ‐irradiated H69 cells specifically harvested 4 days post irradiation were then regarded as senescent H69 cells (s‐H69) and used for subsequent experiments (Figure 2B). Non‐irradiated H69 cells, harvested on the same day (c‐H69), served as controls. Onion skin fibrosis, characterised by concentric layers of fibrosis around the cholangiocyte lining of the bile duct, is a hallmark of PSC, implicating cholangiocytes in the development of fibrosis [25]. Previous studies in co‐culture systems have shown that senescent cholangiocytes exhibiting SASP activate HSCs in a PDGF‐mediated manner [17]. To investigate the expression profile of HSC activation markers in LX‐2 cells activated solely by PDGF‐BB, LX‐2 spheroids were formed in the presence of PDGF‐BB for 24 h. Analysis revealed a significant upregulation of α‐SMA, tissue inhibitor of metalloproteinase‐1 (TIMP‐1), collagen type I alpha 1 (COL1A1) and collagen type III alpha 1 (COL3A1), demonstrating successful HSC activation (Figure 2C). This setup served as a positive control for the subsequent experiment involving c‐H69 or s‐H69 co‐cultured with LX‐2 cells. To investigate whether s‐H69 cells activate LX‐2 cells similarly to PDGF‐BB in 3D culture, we co‐cultured LX‐2 cells with either c‐H69 cells or s‐H69 in a 1:2 ratio (H69:LX‐2) for 24 h, in the form of bi‐cellular spheroids. Notably, we observed a significant upregulation of ACTA2 (up to 33‐fold) and TIMP‐1 in LX‐2 cells when co‐cultured with s‐H69 cells compared to the c‐H69 group. In contrast, the upregulation of COL1A1 and COL3A1 was comparable between the two groups (Figure 2D). These results clearly demonstrate that s‐H69 induces HSC activation in LX‐2 cells when co‐cultured as 3D spheroids. This finding not only highlights the critical role of senescent cholangiocytes in driving HSC activation but also validates our strategy of inducing essential disease phenotypes in the development of our multilineage spheroids for early cholangiopathies.

### 3.3 Cholangiocyte Senescence Drives Hepatic Stellate Cell Activation, Fibrosis and Matrix Remodelling in the s‐H69 Spheroid Model

**FIGURE 3:**
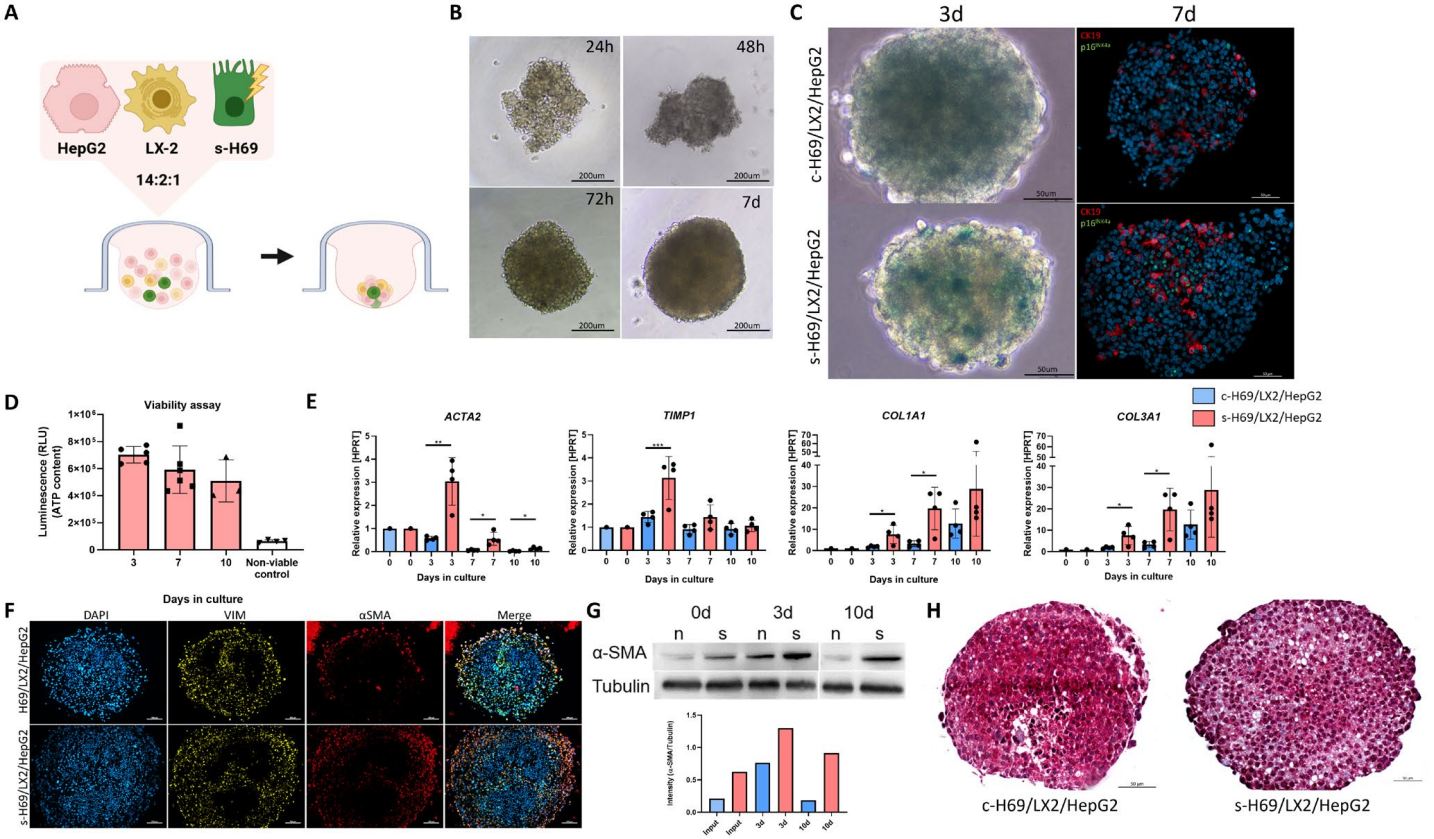
Generation and characterisation of multilineage spheroids containing senescent cholangiocytes and exhibiting fibrotic phenotype. (A) Schematic illustrating the composition and formation process of the s‐H69/LX2/HepG2 spheroid consisting of HepG2, LX‐2 and s‐H69 generated by the hanging drop method. Created with BioRender.com. (B) Bright field microscopy images showing the morphology of s‐H69/LX2/HepG2 spheroids over a 7‐day culture period. (C) Senescence assessment by SA‐β‐gal staining and immunofluorescence. Left: Blue foci indicate SA‐β‐gal activity in s‐H69 spheroids at Days 3 and 7. Right: Immunofluorescence co‐staining for cholangiocyte marker CK19 (red) and senescence marker p16 (green) highlights senescent cholangiocytes within the spheroids after 7 days in culture. Scale bars represent 50 μm. (D) Spheroid viability assessed over time using an ATP‐based assay. Data represent mean ± SD of at least three independent experiments. (E) qPCR analysis of HSC activation and major ECM remodelling markers (ACTA2, COL1A1, TIMP1, COL3A1) in control and senescent spheroids. Expression is shown as fold‐change relative to Day 0 within each group, normalised to HPRT. Each point represents a pool of 10 spheroids; data are mean ± SD of at least three independent experiments (*p < 0.05, ***p < 0.001). (F) Immunofluorescence co‐staining for general HSC marker vimentin (yellow) and HSC activation marker αSMA (red) in control and senescent spheroids, showing increased HSC activation in the latter. Scale bars: 100 μm. (G) Western blot analysis of αSMA expression in control and senescent spheroids at Days 3 and 10, confirming HSC activation predominantly in senescent spheroids. (H) Masson's trichrome staining of Day 10 spheroids. Blue staining indicates collagen deposition in senescent spheroids. Scale bars: 50 μm.

Persistent senescent cholangiocytes, characterised by the SASP, play a pivotal role in activating HSCs and driving fibrosis progression in many cholangiopathies, establishing them as a critical disease phenotype [3, 4, 9, 10]. Building on our previous validation of HSC activation by senescent cholangiocytes in 2D and 3D co‐cultures (Figure 2), we generated multilineage senescent spheroids by incorporating senescent cholangiocytes (s‐H69), activated HSCs (LX‐2), and hepatocyte‐like cells (HepG2) to more comprehensively model cholangiopathy‐associated fibrosis (Figure 3A). Subsequent analysis focused on the overall characteristics and effects on HSC activation within this senescent spheroid. Analysis of these spheroids revealed pronounced SA‐β‐gal activity by Day 3, confirming senescence, and immunofluorescence co‐staining for CK19 and p16 identified senescent cholangiocytes within the senescent spheroids (Figure 3C). Spheroid viability was maintained throughout the culture period, as measured by ATP assays (Figure 3D). Importantly, senescent spheroids showed significant upregulation of HSC activation markers, ACTA2, COL3A1, and TIMP1, at Day 3 compared to controls (Figure 3E). Sustained HSC activation was further confirmed by α‐SMA immunofluorescence and Western blot analyses at Day 10 (Figure 3F,G), and the development of fibrosis was evidenced by Masson's trichrome staining (Figure 3H).

These results demonstrate that the incorporation of senescent cholangiocytes into multilineage spheroids effectively recapitulates key features of cholangiopathy, namely HSC activation and subsequent fibrosis. Thus, the s‐H69/LX‐2/HepG2 spheroid model provides a robust in vitro platform to study senescence‐driven fibrotic processes.

**FIGURE 4:**
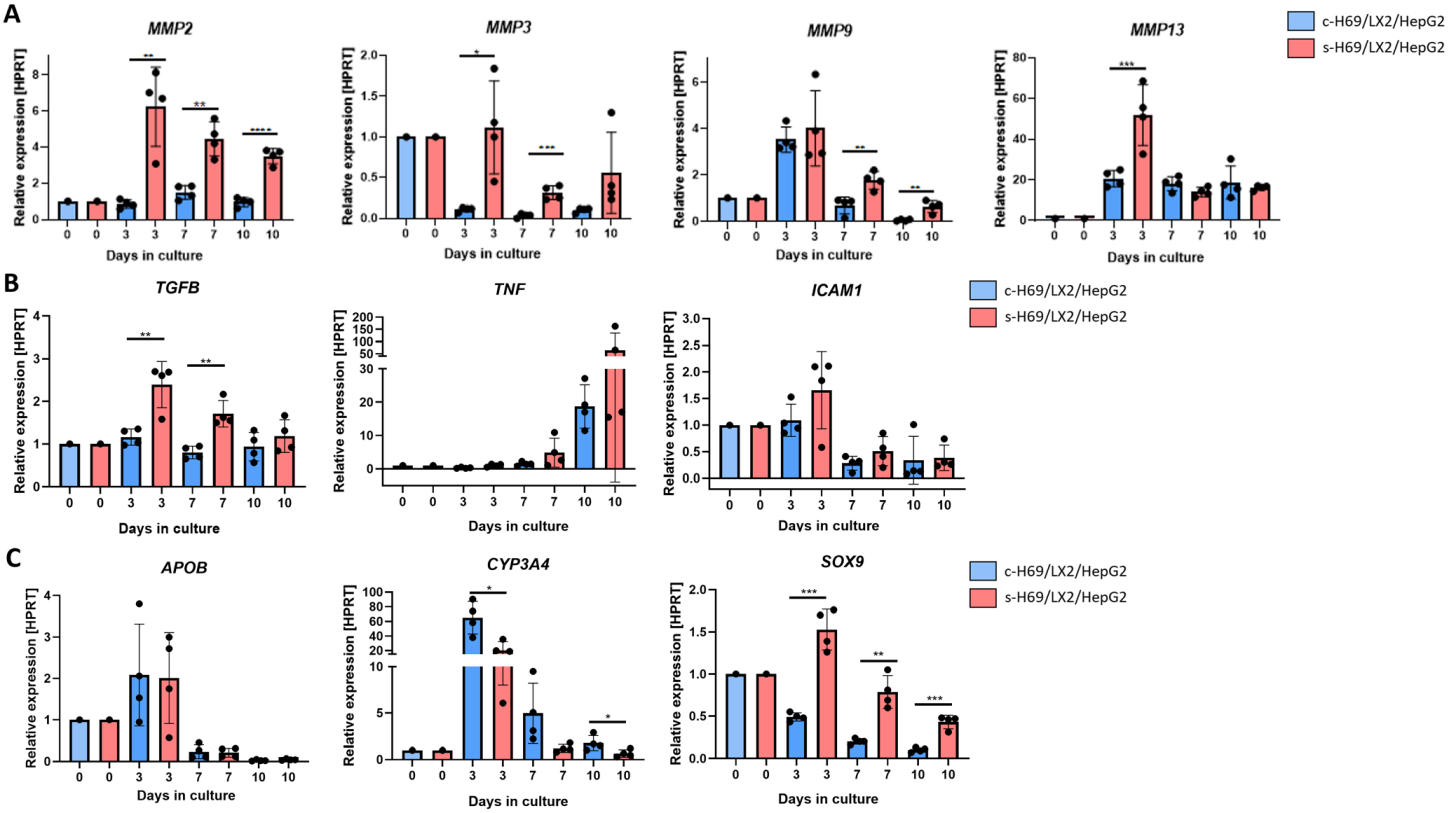
Molecular profiling of matrix remodelling, cytokines, adhesion molecules, and lineage/function‐associated markers in control and s‐H69/LX2/HepG2 spheroids. qPCR analysis of (A) matrix metalloproteinases (*MMP2, MMP3, MMP9, MMP13*); (B) pro‐fibrogenic cytokine (*TGFβ*), adhesion molecule (*ICAM1*), and inflammatory cytokine (*TNF*) and (C) lineage/function‐associated markers including hepatocyte markers *APOB* and *CYP3A4* and cholangiocyte/ductal marker *SOX9*. Data are fold change relative to Day 0 within each group, normalised to *HPRT*. Each data point represents pooled spheroids (*n* = 10). Mean ± SD from at least three independent experiments. Statistical significance: *p < 0.05, **p < 0.01, ***p < 0.001, ****p < 0.0001.

To further characterise fibrogenic and inflammatory responses, we quantified mRNA expression of ECM‐remodelling enzymes, profibrotic cytokines, and lineage/function‐associated markers in control spheroids and senescent spheroids (s‐H69/LX‐2/HepG2) over 10 days of culture (Figure 4).

Senescent spheroids showed significantly higher and earlier upregulation of these ECM‐remodelling enzymes, especially at Day 3. MMP2 upregulation in senescent spheroids was sustained throughout the 10‐day culture period, whereas it remained constant in controls. MMP9 expression peaked initially in both spheroid types but returned to baseline by Day 7 in controls, while remaining approximately twofold elevated in senescent spheroids. MMP3 was markedly downregulated in control spheroids after formation, but in senescent spheroids, it stayed near baseline at Day 3 before decreasing at later time points. MMP13 showed strong upregulation at Day 3 in both groups (approximately 50‐fold in senescent and 20‐fold in control spheroids) and remained stable thereafter (Figure 4A). These patterns suggest dynamic ECM turnover and remodelling in the spheroid cultures. Increased collagen deposition and sustained α‐SMA expression (Figure 3C,E) further characterise the senescent spheroid microenvironment.

*TGFB* expression was higher in senescent spheroids at all time points, with significant upregulation at Days 3 and 7, consistent with its role as a central mediator of HSC activation and fibrosis. *ICAM1*, an adhesion molecule involved in early inflammatory signalling [26, 27], showed a modest increase at Day 3 in senescent spheroids, indicating early induction of inflammatory pathways. TNF expression remained low during early culture but rose sharply by Day 10 in both groups (control ~20‐fold; senescent ~50‐fold), suggesting a delayed pro‐inflammatory response that may reflect later stage inflammatory amplification (Figure 4B).

Hepatocyte‐associated *APOB* and *CYP3A4* exhibited a transient peak in expression at Day 3 in both control and senescent spheroids likely reflecting an early adaptive response to the 3D microenvironment, followed by a decline toward baseline levels. Notably, *APOB* expression was comparable between groups at each time point, while CYP3A4 expression was consistently lower in senescent spheroids, with significant differences at Days 3 and 10 (*p < 0.05), indicating compromised hepatocyte function. Biliary marker *SOX9* was transiently increased at Day 3 in senescent spheroids compared to controls, suggesting early activation of a reactive ductular phenotype. (Figure 4C).

Together, these transcriptional profiles confirm that senescent cholangiocytes accelerate matrix turnover, profibrotic signalling, and early epithelial phenotypic shifts within the 3D multilineage spheroid model. Overall, Figures 3 and 4 demonstrate that senescent cholangiocytes promote hepatic stellate cell activation, fibrosis, and dynamic matrix remodelling in the s‐H69 spheroid model. This highlights the key role of senescence in driving fibrogenic and inflammatory responses characteristic of cholangiopathies.

### 3.4 Application of a Proapoptotic BH3 Mimetic Reduces Fibrosis and Indicates the Potential of Senescent Spheroid for Drug Development

**FIGURE 5:**
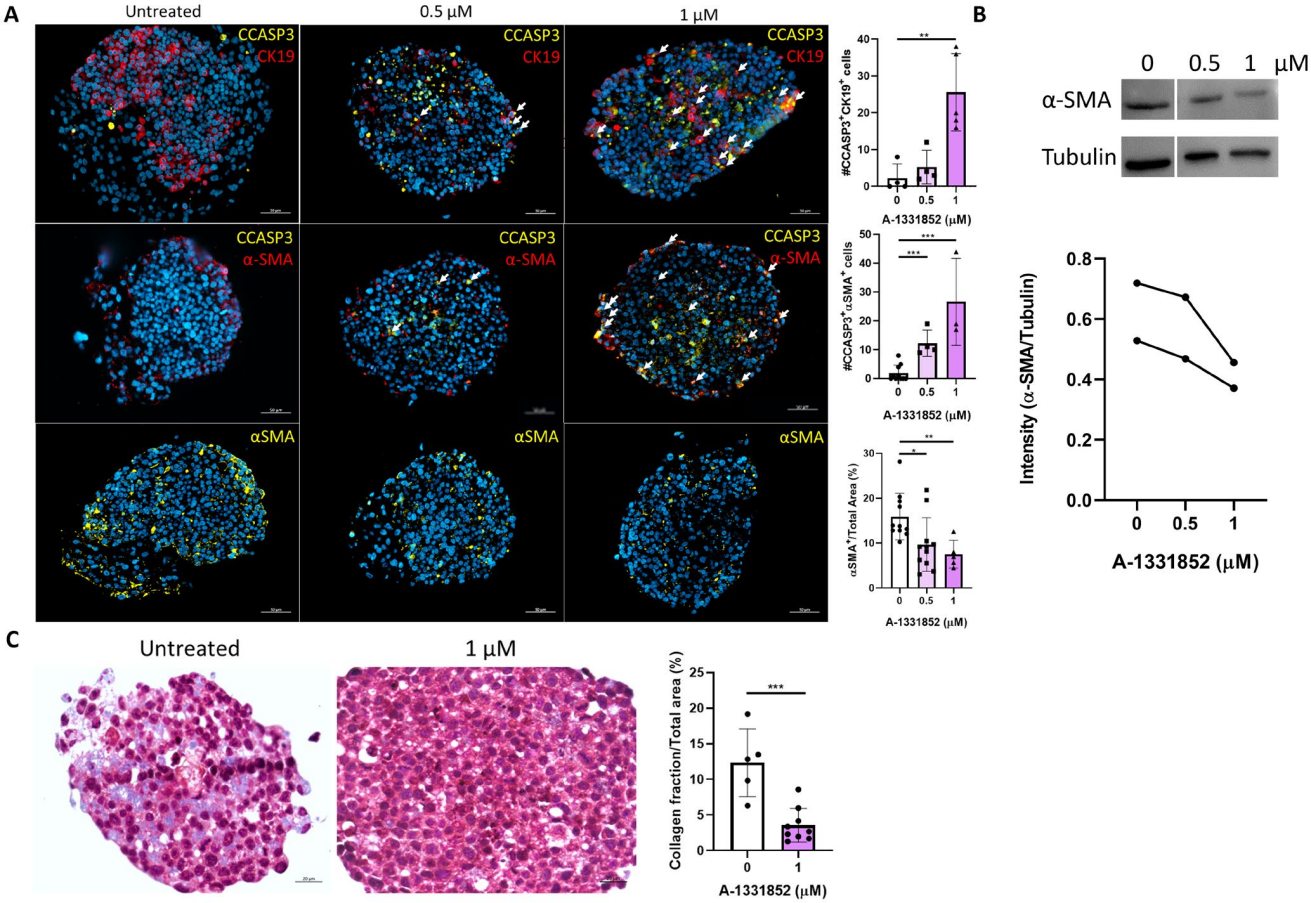
Cholangiocyte apoptosis and downregulation of α‐SMA and collagen in PSC spheroid treated with A‐1331852. (A) Immunofluorescence co‐staining and quantification of cleaved caspase 3 with CK19 and α‐SMA, respectively, on senescent spheroids treated with increasing concentrations of A‐1331852. Single staining for α‐SMA and quantification of the α‐SMA+ area indicate a reduction of α‐SMA+ cells during treatment. Data represent mean ± SD from at least three independent experiments. Statistical significance is indicated as *p < 0.05, **p < 0.01, ***p < 0.001. (B) Western blot for α‐SMA with quantification of two independent analyses normalised to tubulin confirms downregulation after treatment. (C) Masson's trichrome staining and quantification confirm reduced fibrosis (***p < 0.001). Data represent mean ± SD from at least three independent experiments. Statistical significance is indicated as *p < 0.05, **p < 0.01, ***p < 0.001.

Having confirmed the development of senescent spheroid, we investigated its potential as a tool for drug testing. Previous studies have shown that A‐1331852, a pro‐apoptotic BH3 mimetic and Bcl‐xL inhibitor, induces apoptosis in senescent cholangiocytes and α‐SMA‐positive fibroblasts, leading to reduced liver fibrosis in *Mdr2* ^*−/−*^ mice [17]. Hence, we treated the spheroids with two concentrations of A‐1331852 on day three and harvested them on day eight. We investigated the effect on apoptosis in cholangiocytes and activated HSC, α‐SMA expression and fibrosis. Results showed a significant increase in apoptotic cholangiocytes and apoptotic activated HSCs in the spheroids treated with A‐1331852, especially in higher concentration, as demonstrated by immunofluorescence co‐staining of cleaved caspase 3 with CK19 and α‐SMA respectively (Figure 5A). In addition, a reduction in α‐SMA expression was observed by both immunofluorescence staining and Western blot analysis (Figure 5A,B). Furthermore, Masson's trichrome staining revealed a decrease in fibrosis in the treated senescent spheroid compared to untreated controls (Figure 5C). These findings parallel previous observations in Mdr2^−/−^ mice and highlight the potential of spheroids for drug testing. This consistency of therapeutic response across different experimental models underscores the translational relevance of our findings and supports the utility of the senescent spheroid for drug development efforts in cholangiopathies and liver fibrosis.

## 4 Discussion

The aim of this study was to establish a physiologically relevant, reductionist in vitro model to investigate how cholangiocyte senescence drives HSC activation and fibrogenesis. We developed a scaffold‐free 3D multilineage spheroid system incorporating senescent cholangiocytes, HSCs, and hepatocyte‐like cells, enabling controlled analysis of epithelial–stromal crosstalk under senescence‐inducing conditions. By inducing cholangiocyte senescence via irradiation and tracking molecular, structural and functional changes, we show that this model reliably recapitulates early fibrogenic events observed in cholangiopathies. The data demonstrate that senescent cholangiocytes accelerate ECM remodelling, upregulate profibrotic cytokines, alter epithelial phenotypes, and compromise hepatocyte metabolic function.

This reductionist yet physiologically relevant model allows precise induction of cholangiocyte senescence (via irradiation) and the downstream activation of HSCs in a co‐cultured 3D microenvironment. We show that senescent cholangiocytes promote HSC activation and fibrogenesis (*ACTA2*/αSMA and TGFβ upregulation), modulation of immune responses (TGFβ and TNF upregulation), ECM remodelling (MMPs and COL1A1 and COL3A1 upregulations) and collagen deposition (Masson's Trichrome staining), recapitulating early fibrogenic responses (Figures 3 and 4). Treatment with the Bcl‐xL inhibitor A‐1331852 selectively induced apoptosis in both senescent cholangiocytes and activated fibroblasts, reducing fibrosis (Figure 5). These findings are consistent with the therapeutic effects observed in murine models of PSC and PBC and support the model's utility as a platform for senolytic drug screening.

### 4.1 Senescent Cholangiocytes Trigger Robust Fibrotic and Inflammatory Remodelling

Figures 3 and 4 provide the clearest evidence that cholangiocyte senescence acts as a potent upstream driver of fibrogenesis in this system. After assembly, senescent cell clusters were detected at Day 3 and the presence of senescent cholangiocytes was confirmed at Day 7 in the senescent spheroids (Figure 3C). These spheroids exhibited persistent HSC activation, as shown by early and sustained *ACTA2/*α‐SMA expression (Figure 3E–G) and collagen deposition (Figure 3F), coupled with dynamic ECM turnover (Figure 4A). Furthermore, MMP2 remained elevated throughout culture, MMP3 expression was consistently higher than the control, MMP9 upregulation persisted longer than in controls, and MMP13 showed a sharp early induction in both conditions, consistent with an initial remodelling surge. All four MMPs play a critical role in liver fibrosis progression and their increased expression is reported in (cholestasis induced‐) liver fibrosis/inflammation, cirrhosis, HCC and metastasis [28, 29, 30, 31]. The coordinated upregulation of these MMPs alongside collagen and TIMP1 suggests active but imbalanced ECM remodelling. This pattern mirrors early stages of liver fibrosis, where increased matrix degradation coexists with net ECM accumulation, a hallmark of progressive fibrosis rather than resolution. A similar pattern of MMP upregulation alongside collagen and TGFβ induction has been reported in NDMA‐induced liver injury mouse models, where upregulation of TGF‐β1, αSMA and type I collagen and the levels of MMP‐2/MMP‐9 were observed and these effects were attenuated in the absence of MMP13 [32]. Our spheroid system recapitulates these dynamic ECM changes in vitro, suggesting it could serve as a relevant alternative to such animal models for studying early‐stage fibrosis mechanisms.

Functionally, CYP3A4 expression was consistently lower in senescent spheroids compared to controls, indicating impaired hepatocyte metabolic function within a fibrotic microenvironment, whereas APOB levels remained unchanged between the two spheroids (Figure 4C). Together, these transcriptional and phenotypic signatures confirm that senescent cholangiocytes accelerate fibrogenic, inflammatory, and epithelial remodelling processes in a way that closely mirrors early human disease.

### 4.2 Comparison With Ductular Reactions

Our model originally focuses specifically on the senescent cholangiocyte population, rather than reactive ductular cholangiocytes. While the latter are initially proliferative and contribute to early regenerative responses, they may transition into a senescent state under unresolved injury. This ductular to senescent shift marks a pathological inflection point in chronic cholangiopathies, where regeneration fails and fibrotic remodelling begins [1, 11]. Senescent cholangiocytes have been observed in prolonged stages of PSC, PBC, NASH, and chronic viral hepatitis, where their SASP drives immune activation and periductular fibrosis [7, 9, 11, 12] Interestingly, the transient upregulation of SOX9 in senescent spheroids points to early ductular reactions and epithelial plasticity, features commonly seen in chronic cholangiopathies (Figure 4C).

Along with confirmation of senescent cholangiocytes at Day 7 (Figure 3C), these data indicate that the senescent spheroid model contains cholangiocytes with both ductular and senescent characteristics, correlating well with the pathogenesis of multiple cholangiopathies. Although SASP was not fully characterised, early and sustained TGFβ upregulation and dramatic TNF upregulation at Day 10, along with mild ICAM1 induction, suggest initiation of inflammation and immune recruitment (Figure 4B). Undoubtedly, future studies could expand this model by in‐depth cholangiocyte characterisation, comprehensive assessment of cytokine secretion, and investigation of interaction with immune cell co‐culture, to better define the functional and inflammatory signalling networks.

### 4.3 HepG2 Cells as a Hepatic Compartment

The inclusion of HepG2 cells, despite their cancer origin, was essential for robust and reproducible spheroid formation. HepG2 cells are widely used in in vitro liver models due to their metabolic activity, stability, and accessibility, though they do not fully represent primary hepatocytes [19, 33, 34, 35, 36, 37, 38, 39]. In this study, their hepatic identity was validated via albumin expression (Figure 1D), a standard hepatocyte marker [25, 40, 41]. Functionally, *CYP3A4* and *APOB* upregulation on Day 3 in the control spheroids indicated an early adaptive response to the 3D microenvironment. CYP3A4 remained substantially elevated in control spheroids before gradually returning toward baseline by Day 10, consistent with stabilisation of hepatocyte transcriptional activity during spheroid maturation. While additional assays such as CYP activity or urea secretion would strengthen the hepatic readout, our analysis reliably indicates hepatocyte identity within the multicellular system.

### 4.4 Limitations and Opportunities for Refinement

The model reproduces early fibrogenic responses, but several refinements could enhance physiological relevance. Hepatic function was assessed only by APOB and CYP3A4 transcripts; adding CYP activity assays, urea synthesis, and transporter localisation would provide a fuller profile. Broader SASP characterisation beyond TGFβ and TNF would further strengthen mechanistic insight.

HepG2 cells support spheroid viability and structure, while bi‐cellular spheroids (H69 + LX‐2; Figure 2) show HSC activation is driven by senescent cholangiocytes. Future studies could replace HepG2 with primary hepatocytes, HepaRG cells, or add patient‐derived cells for disease‐specific modelling.

Cell‐type proportions were not quantified, which may influence gene expression given the different proliferative state between cell types. However, this mirrors in vivo disease, where a minority of senescent cholangiocytes can exert strong paracrine effects. Future work could use flow cytometry, immunofluorescence, or single‐cell RNA sequencing.

Some lumen‐like structures were observed, but they were not comprehensively studied to confirm organised ducts or periductular fibrosis. Limitations of 2D histological sectioning further restrict interpretation, and 3D imaging would improve characterisation.

The scaffold‐free design remains highly adaptable, enabling integration of additional senescence markers, SASP profiling, cytokine measurements, immune cell co‐cultures, and advanced imaging to explore more complex, disease‐relevant microenvironments.

### 4.5 Therapeutic Implications and Future Applications

The Bcl‐xL inhibitor A‐1331852 selectively eliminated senescent cholangiocytes and activated HSCs, reducing fibrosis, consistent with murine PSC and PBC studies [9, 18] (Figure 5). This highlights the model's potential for senolytic drug testing and antifibrotic compound screening. The platform is adaptable to high‐throughput formats, enabling parallel therapeutic testing. Beyond PSC and PBC, it could model other senescence‐associated cholangiopathies, such as post‐cholangitis cirrhosis and drug‐induced cholestasis, and be expanded to investigate immune, metabolic, and transporter‐related mechanisms in fibrotic liver disease.

## 5 Conclusion

We present a reproducible, physiologically relevant human 3D multilineage spheroid model that recapitulates the fibrogenic cascade initiated by cholangiocyte senescence. This scaffold‐free system captures key pathological features including cholangiocyte senescence, HSC activation, and ECM deposition within a controlled microenvironment. The model enables senolytic drug testing, providing a scalable and reproducible platform for preclinical screening. By bridging the gap between over‐simplified 2D models and complex animal models, our senescent spheroid system offers a versatile tool for investigating early‐stage cholangiopathies and supports the development of targeted antifibrotic and anti‐senescent therapies.

## Author Contributions

**Cheuk‐Ting Wu:** conceptualisation, investigation, methodology, formal analysis, validation, visualisation, data curation, writing original draft, review and editing. **Anja Moncsek:** conceptualisation, supervising the project and detailed methodology, funding acquisition, writing review and editing. **Joachim Mertens:** conceptualisation, supervising the project and methodology, funding acquisition, writing review and editing.

## Conflicts of Interest

The authors declare no conflicts of interest.

## Supporting information


**Data S1:** liv70352‐sup‐0001‐Supinfo.docx.

## Data Availability

The raw and processed data required to reproduce these findings cannot be shared at this time due to technical or time limitations.
