# Supplementary material for: Development and Validation of a Scaffold‐Free Human Multilineage Spheroid Model for Early Stage Cholangiopathies Driven by Cholangiocyte Senescence
Source: Liver Int. 2025 Sep 10;45(10):e70352. doi: 10.1111/liv.70352 (PMC12423616; doi:10.1111/liv.70352)
Supplement: Supplementary file 1 — Data S1: liv70352‐sup‐0001‐Supinfo.docx. [file LIV-45-0-s001.docx]

**Supplementary Data**

**Table 1. Antibody used in IF analysis.**

**Primary antibodies**

| **Protein** | **Species** | **Supplier** | **Catalog number** | **Used concentration** |
| --- | --- | --- | --- | --- |
| Albumin | Mouse | Santa Cruz | sc-271605 | 1/500 |
| α-SMA | Rabbit | Cell Signaling | 48938S | 1/200 |
| α-SMA | Rabbit | abcam | 124964S | 1/500 |
| Cleaved Caspase-3 | Rabbit | Cell Signaling | #9661 | 1/500 |
| Cytokeratin 19 | Mouse | Santa Cruz | sc-374192 | 1/500 |
| Ki67 | Rat | ThermoFisher Scientific | 14-5698-82 | 1/500 |
| RIPK3 | Mouse | Bio-Techne / R&D | MAB7604-SP | 1/500 |
| Vimentin | Rabbit | Abcam | ab92547 | 1/500 |

**Secondary antibodies:**

| **Species and conjugated fluorophore** | **Supplier** | **Catalog number** | **Used concentration** |
| --- | --- | --- | --- |
| Goat-anti-rabbit, Alexa Fluor 594 | ThermoFisher Scientific | A-11012 | 1/150 |
| Goat-anti-mouse, Alexa Fluor 647 | ThermoFisher Scientific | A-21235 | 1/150 |
| Goat-anti-rat,  Alexa Fluor 647 | ThermoFisher Scientific | A-21247 | 1/150 |
| DAPI | Carl Roth | 6335 | 1/1000 |

**Table 2. Antibody used in Western Blot analysis**

**Primary antibodies:**

| **Protein** | **Species** | **Supplier** | **Catalog number** | **Used concentration** |
| --- | --- | --- | --- | --- |
| α-SMA | Rabbit | abcam | 124964S | 1/10000 |
| Tubulin | Mouse | abcam | Ab44928 | 1/80 |

**Secondary Antibodies:**

| **Species and conjugate** | **Supplier** | **Catalog number** | **Used concentration** |
| --- | --- | --- | --- |
| mouse anti rabbit IgG-HRP | Santa Cruz | sc-2357 | 1/1000 |
| goat anti mouse IgG-HRP | Santa Cruz | sc-2005 | 1/1000 |
